# Library Construction from Subnanogram DNA for Pelagic Sea Water and Deep-Sea Sediments

**DOI:** 10.1264/jsme2.ME17132

**Published:** 2017-11-28

**Authors:** Miho Hirai, Shinro Nishi, Miwako Tsuda, Michinari Sunamura, Yoshihiro Takaki, Takuro Nunoura

**Affiliations:** 1 Research and Development (R&D) Center for Marine Biosciences, Japan Agency for Marine-Earth Science and Technology (JAMSTEC) 2–15 Natsushima-cho, Yokosuka 237–0061 Japan; 2 Ecosystem Observation and Evaluation Methodology Research Unit, Project Team for Development of New-generation Research Protocol for Submarine Resources, Japan Agency for Marine-Earth Science and Technology (JAMSTEC) 2–15 Natsushima-cho, Yokosuka 237–0061 Japan; 3 Department of Earth and Planetary Science, The University of Tokyo Tokyo Japan; 4 Department of Subsurface Geobiological Analysis and Research, Japan Agency for Marine-Earth Science and Technology (JAMSTEC) 2–15 Natsushima-cho, Yokosuka 237–0061 Japan

**Keywords:** metagenomics, subnanogram DNA, marine microbiome

## Abstract

Shotgun metagenomics is a low biased technology for assessing environmental microbial diversity and function. However, the requirement for a sufficient amount of DNA and the contamination of inhibitors in environmental DNA leads to difficulties in constructing a shotgun metagenomic library. We herein examined metagenomic library construction from subnanogram amounts of input environmental DNA from subarctic surface water and deep-sea sediments using two library construction kits: the KAPA Hyper Prep Kit and Nextera XT DNA Library Preparation Kit, with several modifications. The influence of chemical contaminants associated with these environmental DNA samples on library construction was also investigated. Overall, shotgun metagenomic libraries were constructed from 1 pg to 1 ng of input DNA using both kits without harsh library microbial contamination. However, the libraries constructed from 1 pg of input DNA exhibited larger biases in GC contents, *k*-mers, or small subunit (SSU) rRNA gene compositions than those constructed from 10 pg to 1 ng DNA. The lower limit of input DNA for low biased library construction in this study was 10 pg. Moreover, we revealed that technology-dependent biases (physical fragmentation and linker ligation vs. tagmentation) were larger than those due to the amount of input DNA.

Shotgun metagenomic sequencing has become an indispensable technique in environmental microbiology owing to the advancement of high-throughput sequencing technologies. This technique enables us to assess the microbial diversity and potential functions of microbial communities and sometimes provides opportunities to obtain almost complete genome sequences or certain microbes (26, 27). However, the limitations of sample volumes, a low biomass, and/or the presence of a high concentration of humic substances complicate the application of shotgun metagenomics to deep-sea environments such as bathypelagic to hadal waters, benthic habitats under oligotrophic oceans, and subseafloor sediments (12, 16, 20, 34).

If the amount of DNA is insufficient to construct a shotgun metagenomic library, whole genome amplification (*e.g.*, multiple displacement amplification [MDA] using Phi29 DNA polymerase [1, 7]) may be applied to obtain a sufficient amount of DNA prior to library construction. However, uneven amplification, negative biases against high GC templates, and chimera formation have been reported in MDA technology (13, 22, 24, 33). As a low biased method for shotgun metagenomic library construction from no more than 1 ng DNA, PCR-based library construction methods have been applied to environmental samples. One method involves the combination of physical fragmentation and sequencing adaptor ligation, while the other consists of the simultaneous fragmentation and tagging of a sequencing adaptor (“tagmentation”) using a transposase prior to PCR amplification (9, 23, 28). Successful low biased shotgun metagenomic library construction with as little as 100 fg of DNA was recently reported using a tagmentation technique with the Nextera XT DNA Library Preparation Kit (23). In comparisons of the two methods, the tagmentation technique was found to introduce a lower amplification bias in GC contents, as described previously (11, 14), but has the disadvantages of shorter individual contig and total contig lengths (11). In addition, the combination of physical fragmentation and sequencing adaptor ligation for some of the classical library construction kits requires more time and special techniques (23).

However, in these studies, prokaryotic or viral DNA from mock and/or marine planktonic communities was used as the testing material. Thus, the effects of chemical contaminants on environmental DNA solutions in shotgun metagenomic library construction were not examined, despite the issue of chemical contamination in environmental DNA solutions, such as humic acids and polysaccharide and phenolic compounds, on PCR reactions (30, 32, 34). In addition, new library construction kits for low-input DNA based on adaptor ligation with a low amplification bias have been developed, including the KAPA Hyper Prep Kit, and require shorter working times and fewer special techniques (5). We herein examined the applicability of shotgun library construction kits based on the adaptor ligation technique, such as the KAPA Hyper Prep Kit and Ovation SP+ Ultralow Library Systems, and that based on the tagmentation Nextera XT DNA Library Preparation Kit for the low input of environmental DNA (1 pg to 1 ng) extracted from pelagic seawater and deep-sea sediments.

## Materials and Methods

### Sample collection

Upper bathyal marine sediment was taken from the area offshore of Tohoku in Honshu Island, Japan (station M-840, 38°30.00′, 142°20.31′, water depth of 838 m) using a multiple coring system during the Japan Agency for Marine-Earth Science and Technology (JAMSTEC) *RV Mirai* MR12-E02 cruise (March 2012) (17). Abyssal marine sediment was taken from the north slope of the Challenger Deep of the Mariana Trench (station A2, 11°44.78 N, 142°6.52 E, water depth of 5,838 m) using a multiple coring system during the JAMSTEC *RV Kairei* KR14-01 cruise (January 2014). A sediment core was subsampled onboard at 2-cm intervals from the top to 10 cm below the seafloor (cmbsf) using spatulas. These sediment samples were stored at −20 or −80°C. Subarctic surface water was taken offshore of Tohoku, Japan (station 22, 38°45.08N, 146°18.45E, water depth of 0 m) using a bucket during the JAMSTEC *RV Mirai* MR14-04 cruise (July 2014). Microbial cells in approximately 3.5 L of seawater were filtered using a cellulose acetate membrane filter (pore size of 0.22 μm, diameter of 47 mm) (Advantec, Tokyo, Japan). The filter was stored at −80°C.

### DNA extraction and purification from deep-sea sediments

Approximately 5 g of the frozen sediment sample at the uppermost section (0–2 cmbsf) was used for DNA extraction. Total DNA was extracted using a Power Max Soil DNA Isolation Kit (MoBio Lab., Carlsbad, CA, USA). The resultant DNA solution was concentrated by ethanol precipitation and eluted in 200 μL DNase and RNase-Free TE Buffer (NIPPON GENE, Tokyo, Japan).

Further purification was performed using the magnetic bead purification system, MagExtractor-PCR & Gel Clean Up (TOYOBO, Osaka, Japan) (19). The quality of purified DNA was assessed using a NanoDrop^TM^ 2000 spectrometer (Thermo Fisher Scientific, Waltham, MA, USA) at an absorbance ratio of A260/280 nm. A DNA LoBind tube (Eppendorf, Hamburg, Germany) was used for the storage of environmental DNA to minimize DNA adsorption on the plastic tube. All the metagenomic libraries sequenced in this study were constructed from the DNA assemblages purified with a magnetic bead purification system.

All extraction and purification processes for DNA, except centrifugation, were performed at a clean bench or under the open clean system KOACH T 500-F (KOKEN, Tokyo, Japan). The amount of DNA was quantified using an Invitrogen Qubit-fluorometer (Thermo Fisher Scientific). Purified DNA was stored at −80°C until PCR amplification and metagenomic library construction.

### DNA extraction and purification from a water sample

Half of the frozen filter with microbial cells was minced to smaller than 5 mm^2^ on an aseptic plastic Petri dish. Minced pieces of the filter were collected in a 2-mL DNA LoBind tube. The microbial cells were statically lysed in 400 μL of FL Buffer (400 mM Tris-HCl [pH 8.0], 60 mM EDTA, 150 mM NaCl and 1% [wt/v] SDS) at 60°C for 10 min. The reaction was stopped by the addition of 120 μL of 3 M potassium acetate buffer (pH 4.8) and incubated on ice for 5 min. FL Buffer and 3 M potassium acetate buffer (pH 4.8) were prepared in NIPPON GENE with our custom order. The supernatant obtained by centrifugation (15,000 rpm at room temperature for 5 min) was transferred to a 1.5-mL DNA LoBind tube, and centrifugation was repeated. Environmental DNA in the supernatant was further purified using a Power Soil DNA Isolation Kit (MoBio Lab) identical to the DNeasy PowerSoil Kit (QIAGEN, Hilden, Germany). The supernatant was mixed with a double volume of C4 solution in the Power Soil DNA Isolation Kit. After this step, DNA was purified following the manufacturer’s manual for the DNA isolation kit. Fifty microliters of DNA solution was obtained and purified DNA was stored in a 1.5-mL DNA LoBind tube. Extracted DNA was further purified using a magnetic bead purification system as described above, and was used for the construction of the metagenomic libraries sequenced in this study.

### Metagenomic library construction and sequencing

A KAPA Hyper Prep Kit (for Illumina) (KAPA Biosystems, Wilmington, MA, USA), Nextera XT DNA Library Preparation Kit (Illumina, San Diego, CA, USA), and Ovation SP+ Ultralow Library System (NuGEN, San Carlos, CA, USA) were used for metagenomic library construction (Fig. S1). Environmental DNA was diluted to a certain concentration with DNase and RNase-Free Water (NIPPON GENE) prior to shotgun metagenomic library construction. Prior to PCR amplification for sequence library construction, all bottled water and sample tubes used for shotgun library construction were processed under UV light irradiation at a clean bench and opened under the open clean system KOACH T 500-F to minimize microbial and nucleotide contamination.

The library constructed from 1 ng of DNA was created following the manufacturers’ protocols. Minor modifications were made to the protocols of the KAPA Hyper Prep Kit (KAPA HP) and Nextera XT DNA Library Preparation Kit (Nextera XT) for library construction from less than or equal to 100 pg of DNA, as described below. The Ovation SP+ Ultralow Library System (Ovation SP+) was worked in the Mondrian^TM^ SP+ Workstation.

In order to prepare the starting DNA for KAPA HP and Ovation SP+, Covaris S220 (Woburn, MA, USA) was used for physical DNA fragmentation using the conditions described below to obtain a peak fragment size: 400 bp; Peak Power: 175.0, Duty Factor: 5.0, Cycles/Burst: 200, and Time: 55 s. In the KAPA HP kit, each 110 μL of the adaptor ligation reaction mixture consisted of 60 μL of input DNA, 5 μL of adaptor stock, 5 μL of water, 30 μL of ligation buffer, and 10 μL of DNA ligase. The adaptor stock concentration was diluted to 0.3 to 150 nM to examine the best adaptor concentration for library construction with 1 to 100 pg DNA. PCR cycles applied to KAPA HP were 14, 18, 21, and 25 cycles for 1,000, 100, 10, and 1 pg DNA, respectively. In Ovation SP+, 11 cycles of PCR were conducted for 1,000 pg DNA following the manufacturers’ recommendations.

The amplicon tagment mix (ATM) in Nextera XT, which includes the enzyme used for tagmentation, was diluted 1:10 in nuclease-free water for library construction using 1 to 100 pg input DNA following Rinke *et al.* (23). Each 20 μL of the tagmentation reaction mixture consisted of 10 μL TD buffer, 5 μL of input DNA, and 5 μL of diluted ATM. In 1 ng input DNA, original ATM was used for the reaction mixture. PCR cycles applied for library construction were 12, 14, 17, and 20 cycles for 1,000, 100, 10, and 1 pg DNA, respectively, following Rinke *et al.* (23) and the manufacturer’s protocol. The manufacturer recommends 12 cycles of the PCR reaction for no less than 1 ng input DNA.

Amplified libraries were purified using Agencourt AMPure XP (Brea, CA, USA). The quality of the purified libraries was assessed using an Agilent High Sensitivity DNA Kit on an Agilent 2100 Bioanalyzer (Santa Clara, CA, USA). The sequencing libraries were further quantified using the KAPA Library Quantification Kit. Metagenomic libraries were mixed with PhiX Control v3 (Illumina) at a ratio of 9:1 and sequenced with an Illumina MiSeq Reagent Kit v3 (600 cycles).

### Data processing for metagenomic libraries

Metagenomic reads were subjected to adaptor clipping and quality trimming using Trimmomatic v0.33 (3) with the following parameters: “ILLUMINACLIP:TruSeq3-PE-2.fa:2:30:10:8:true LEADING:3 TRAILING:3 SLIDINGWINDOW:4:20 MINLEN:100”. The first three nucleotides with quality scores less than 20 were cut from the 3′ and 5′ read ends. Reads were processed using a sliding window method, cutting once the average quality within the window (4 base) fell below the threshold (Q20). Reads with a length of fewer than 100 nucleotides were then removed. Low-complexity reads was filtered out using PRINSEQ version 0.20.4 (25). PCR duplicates were removed with Picard version 2.8.0 (http://broadinstitute.github.io/picard). The processed high-quality and clean reads in each library were used in subsequent analyses.

In the community analysis based on metagenomic sequences, small subunit (SSU) rRNA gene sequences were identified using Metaxa2 software (2). Taxonomy assignments were performed based on the results of a BLAST search against the SILVA123 database (31), using the MEGAN program (10) with the following settings: Min Support 1, Min Score 50, Max Expected 1e-5, Top Percent 10.0. SSU rRNA gene community compositions were compared among the metagenomic libraries. The genera typically found in soil and the human body (and often detected in previous negative control PCR experiments in our laboratory)—such as *Bradyrhizobium*, *Brevundimonas*, *Burkholderiaceae*, *Delftia*, *Erythrobacter*, *Lactococcus*, *Legionella*, *Methylobacterium*, *Mycobacterium*, *Neisseria*, *Novosphingobium*, *Propionibacterium*, *Sphingobium*, *Sphingomonas*, *Sphingopyxis*, *Staphylococcus*, *Stenotrophomonas*, and *Streptococcus*—were recognized as potential contaminants (Table S1) (21). Contaminants were excluded from subsequent cluster analyses for SSU rRNA gene community compositions.

### SSU rRNA gene tag sequencing

SSU rRNA gene fragments were amplified with LA Taq polymerase (TAKARA Bio, Kusatsu, Japan) using the universal primer set of U530F and U907R (18) from all three environmental DNA assemblages. The Illumina adaptor sequence (ACACTCTTTCCC TACACGACGCTCTTCCGATCT) and Illumina Multiplexing PCR Primer 2.0 sequence (GTGACTGGAGTTCAGACGTGTGC TCTTCCGATCT) were added at the 5′ ends of the primers U530F and U907R, respectively. The PCR reaction mixture was prepared as follows: LA Taq polymerase (final conc. 0.1 unit μL^−1^), GC I Buffer, dNTP (final conc. 0.25 mM each), primers (final conc. 0.2 μM each), and template DNA. PCR amplification conditions consisted of denaturation at 96°C for 1 min and 25 to 35 amplification cycles; denaturation at 96°C for 25 s, annealing at 52°C for 45 s, and elongation at 72°C for 1 min. PCR reaction mixtures were prepared at a clean bench or under an open clean system, as described above. After PCR amplification, excess dNTP and oligonucleotide primers in the PCR reaction mixture were digested by Exonuclease I (Affymetrix, Santa Clara, CA, USA) and Shrimp alkaline phosphatase (Affymetrix). A 100-fold dilution of the reaction mixture was then used as a template for the next PCR reaction with Ex Taq polymerase (TAKARA Bio) to ligate the sequencing index and adaptor sequences. In this PCR reaction, the P5 primer consisted of the TruSeq HT Kit D501–D508 Adaptor sequence and P5 indexes (AATGATACGGCGACCACCGAGATCTACAC [7 bp of the P5 index] ACACTCTTTCCCTACACGAC), and the P7 primer consisted of the 5′ region of TruSeq Index PCR Primer and P7 indexes (CAAGCAGAAGACGGCATACGAGAT [7 bp of the P7 index] GTGACTGGAGTTCAGACGT). The P5 and P7 indexes constructed in this study are shown in Table S2. The PCR reaction mixture was prepared as follows: EX Taq polymerase (final conc. 0.025 unit μL^−1^), Ex Buffer (Mg^+^), dNTP (final conc. 0.2 mM each), primers (final conc. 0.4 μM each), and template DNA solution (5% of total volume). PCR amplification conditions consisted of denaturation at 96°C for 1 min and 15 amplification cycles; denaturation at 96°C for 30 s, annealing at 65°C for 45 s, and elongation at 72°C for 1 min. The amplified library was purified with Agencourt AMPure XP twice and quantified using the KAPA Library Quantification Kit. The amplicon library was sequenced with PhiX Control v3 at a ratio of 1:1 using the Illumina MiSeq Reagent Kit v3 (600 cycles).

The sequence adaptor sequence and low-quality reads of the SSU rRNA sequence reads were clipped and trimmed, respectively, using Trimmomatic v0.33 (3) with the parameters for the processing of the shotgun metagenomic reads as described above. PCR primers were removed from the processed sequences using Cutadapt v1.10 (15). The forward sequences of each library were analyzed using QIIME v1.5.0 pipelines (4). Briefly, after removing potential chimera sequences, the sequences were clustered into operational taxonomic units (OTUs) with 97% sequence identity in each library. The taxonomic position of each OTU was automatically assigned based on the BLAST analysis using the SILVA123 database (31) as a reference data set of SSU rRNA genes. Sequences closely related to the potential laboratory contaminants described above were omitted from further analyses (Table S3).

### Comparison of sequence libraries

The 11-nt *k*-mers were counted using the shortKmerCounter.jar program, and dissimilarities between metagenomic libraries were calculated using the custom R script of the “kmer project” software package (8). Bray-Curtis dissimilarity matrices among the shotgun metagenomic libraries were calculated from the *k*-mer profiles using the vegan package in the R-environment (http://www.R-project.org). The matrices were applied to the hierarchical clustering of the hclust function in R. Cluster analyses of SSU rRNA gene communities among the shotgun metagenomic and tag sequencing libraries were executed with Bray-Curtis dissimilarity matrices based on taxonomic counts. A non-metric multidimensional scaling (NMDS) analysis was performed based on the Bray-Curtis distance matrix using the function metaMDS from the vegan package in R.

### Accession number

The data set obtained in this study was deposited with the accession number DRA005726.

## Results

### Effects of chemical contaminants on shotgun metagenomic library construction

In order to examine the effects of the chemical contaminants of an environmental DNA solution on shotgun metagenomic library construction using KAPA HP and Nextera XT, three types of environmental DNA assemblages were prepared. Surface seawater was obtained from the subarctic North Pacific, high total organic carbon (TOC) upper bathyal sediment (3.6%) (17) was taken from the eutrophic pelagic Northwest Pacific, offshore of Tohoku, Japan, and low TOC (0.35%) abyssal sediment was sampled from the north slope of the Mariana Trench (Nomaki *et al.* unpublished).

Metagenomic libraries were successfully constructed from 1 ng of DNA extracted from the low TOC abyssal sediment using both library construction kits. However, we were unable to construct shotgun metagenomic libraries from the DNA extracted from subarctic surface water using KAPA HP or the high TOC upper bathyal sediment using Nextera XT (Fig. S2).

### Shotgun metagenomic library construction from no more than 1 ng environmental DNAs

Since unsuccessful library construction was observed in several cases, all environmental DNA assemblages were further purified using a magnetic bead purification system applicable to DNA lengths between 60 bp and 50 kb. The ratio of absorbance at 260 and 280 nm of DNA was higher than 2.0 after magnet bead purification, while that before magnet bead purification was approximately 1.7. As a result, shotgun metagenomic libraries were successfully constructed using KAPA HP and Nextera XT from 1 ng DNA from all tested samples following the manufacturers’ protocols (Fig. S3 and Table 1). Thus, we further examined sequence library construction using kits from 1 to 100 pg of the three environmental DNA assemblages purified with a magnetic bead purification system.

Following the modified protocol of Nextera XT (23), metagenomic libraries were successfully constructed using 1 to 100 pg of the three environmental DNA assemblages (Fig. S3). In KAPA HP, adaptor stock concentrations that maintain adaptor: insert molar ratios of 200:1 are recommended for 1 to 50 ng of DNA by the manufacturer’s protocol. Thus, 300 nM of adaptor stock solution was prepared for 1 ng DNA in this study. However, an adaptor:insert molar ratio of 200:1 did not function for 1 or 10 pg of environmental DNA (data not shown). Thus, we examined library construction with a higher adaptor:insert molar ratio for a lower amount of starting DNA; at adaptor stock concentrations of 150 nM for 100 pg of DNA (a ratio of 1,000:1), 30 and 150 nM for 10 pg of DNA (2,000:1 and 10,000:1, respectively), and 5 and 150 nM for 1 pg of DNA (3,300:1 and 100,000:1, respectively) (Fig. S4). As a result, we successfully obtained sufficient DNA for metagenomic libraries from 1 to 100 pg of DNA to sequence on MiSeq. The best library yield was obtained at an adaptor stock concentration of 150 nM for 1 to 100 pg of DNA (Fig. S4). We sequenced all metagenomic libraries obtained using 1 pg to 1 ng of DNA from the three samples and library construction kit. In addition, two libraries constructed with Ovation SP+ from 1 ng of deep-sea sediment samples was also sequenced (Fig. S3 and Table 1).

### Evaluation of sequence reads

The read quality of each shotgun metagenomic library is summarized in Table 1. Low-complexity and duplication reads in KAPA HP libraries (0.01 to 0.4% and 0.02 to 33.5%, respectively) were generally more abundant than those in Nextera XT libraries (<0.03% and 0.01 to 5.1%, respectively). The abundance of duplication reads increased with decreasing input DNA in the KAPA HP and Nextera XT kits. The sequence reads of the libraries constructed from 1 pg DNA using KAPA HP harbored a higher abundance of low-quality reads (34.4 to 55%) than those of Nextera XT libraries (10.8 to 15.1%).

**Fig. 1:**
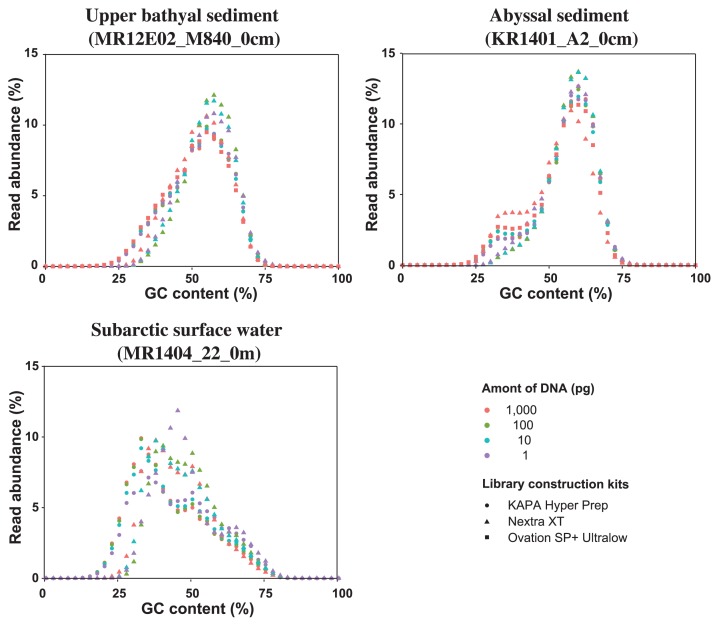
GC distribution of MiSeq reads for each metagenomic library constructed from environmental DNA assemblages extracted from upper bathyal sediment (MR12E02_M840_0cm), abyssal sediment (KR1401_A2_0cm), and subarctic surface water (MR1404_22_0m).

The average read GC content of the processed reads indicated that the deep-sea sediment DNA assemblages were dominated by GC-rich genomes, whereas the surface water DNA assemblage was dominated by AT-rich genomes (Fig. 1 and Table 1). KAPA HP and Ovation SP+ based on the physical fragmentation and linker ligation method presented similar GC contents and GC read abundance profiles. The libraries constructed with tagmentation-based Nextera XP presented a higher average read GC content and peak positions (GC%) of read abundance (*e.g.* 54–57% and 55.5–59.5% in MR12E02_M840_0cm libraries) than those constructed with KAPA HP and Ovation SP+ (52.3–53.7% and 56.5–57.5%) (Table 1). Average read GC contents were significantly different between Nextera XT and KAPA HP libraries from the same amount of starting DNA (*P*-values, <0.001 in Welch’s t-test with sample sizes of one million reads). The read abundance pattern of high GC reads was generally similar among the libraries, whereas the coverage of AT-rich genome fragments, particularly >70% in Nextera XT libraries, differed from those in the other kits. We also observed variations among the libraries, depending on the amount of starting DNA. The average read GC contents of the Nextera XT libraries constructed from 1 to 100 pg DNA were higher than those constructed from 1 ng DNA (*P*-values, <0.001 in Welch’s t-test with sample sizes of one million reads), whereas this was not apparent in the KAPA HP libraries (Table 1). In the two deep-sea sediment libraries, the peak of Nextera XT libraries constructed from 1 to 100 pg DNA shifted to a higher GC content than that constructed from 1 ng DNA. In the abyssal sediment libraries (KR1401_A2_0cm libraries), the minor peak of AT-rich reads (35.5 to 37.5% GC content) was absent in the Nextera XT libraries constructed from 1 to 100 pg DNA. In the subarctic surface water libraries (MR1404_22_0m libraries), the read abundance pattern of the Nextera XT library constructed from 1 pg DNA was different from those in the Nextera XT libraries from the other input DNA.

**Fig. 2:**
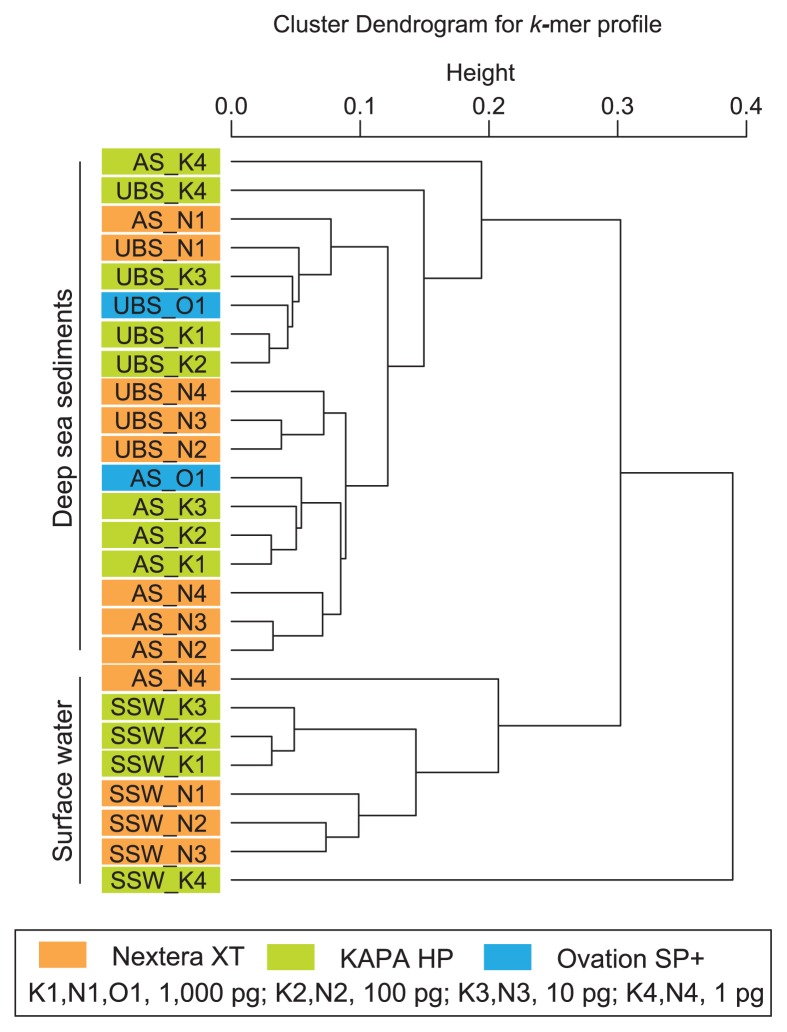
A *k*-mer clustering analysis of MiSeq reads for each metagenomic library constructed from environmental DNA assemblages extracted from upper bathyal sediment (UBS: MR12E02_M840_0cm), abyssal sediment (AS: KR1401_A2_0cm), and subarctic surface water (SSW: MR1404_22_0m).

We also conducted a *k*-mer clustering analysis to compare metagenomic libraries (Fig. 2). Among the libraries constructed from 10–1,000 pg DNA, the *k*-mer feature was primarily defined by the source DNA, and was secondarily influenced by the library construction methods (physical fragmentation and linker ligation vs. tagmentation). The positions of the KAPA HP libraries constructed from 1 pg DNA were distinct from those constructed from 10 to 1,000 pg DNA (Fig. 2). In addition, the Nextera XT library constructed from 1 pg DNA of subarctic surface water (SSW_N4) presented unique *k*-mer features from the other Nextera XT libraries constructed from larger amounts of the same starting DNA.

### Composition of SSU rRNA gene sequences

**Fig. 3:**
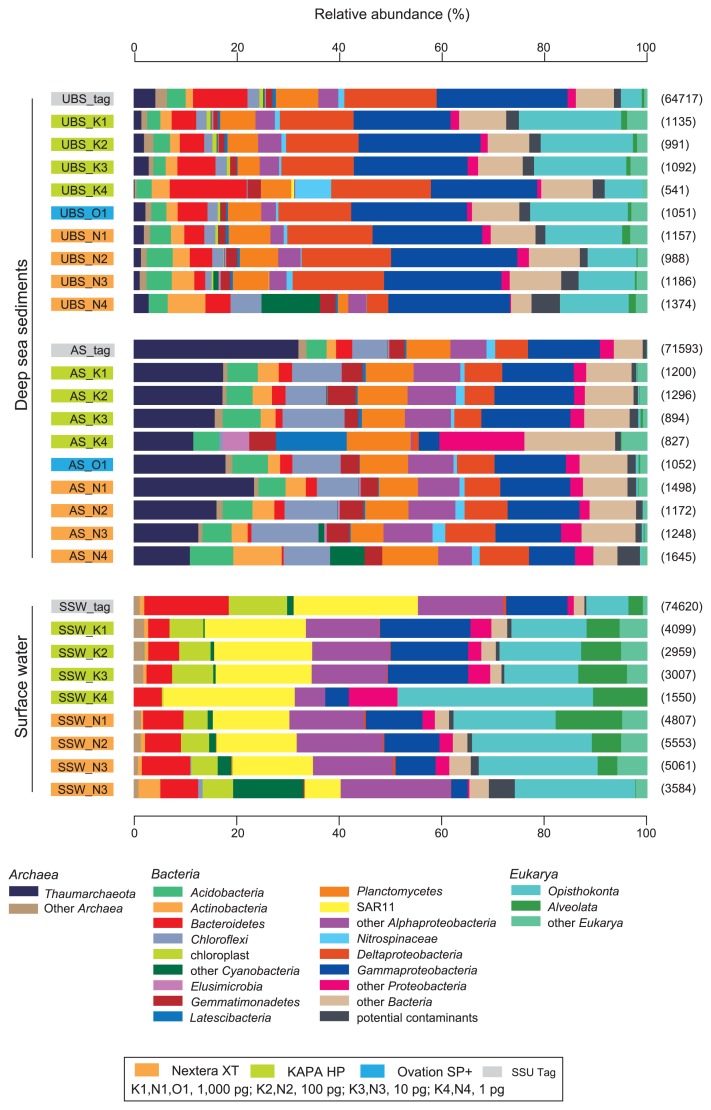
Compositions of SSU rRNA gene tag communities and SSU rRNA gene sequence communities identified from metagenomic libraries obtained from environmental DNA assemblages extracted from upper bathyal sediment (UBS: MR12E02_M840_0cm), abyssal sediment (AS: KR1401_A2_0cm), and subarctic surface water (SSW: MR1404_22_0m). Numbers in parentheses indicate the number of sequences identified in each library.

**Fig. 4:**
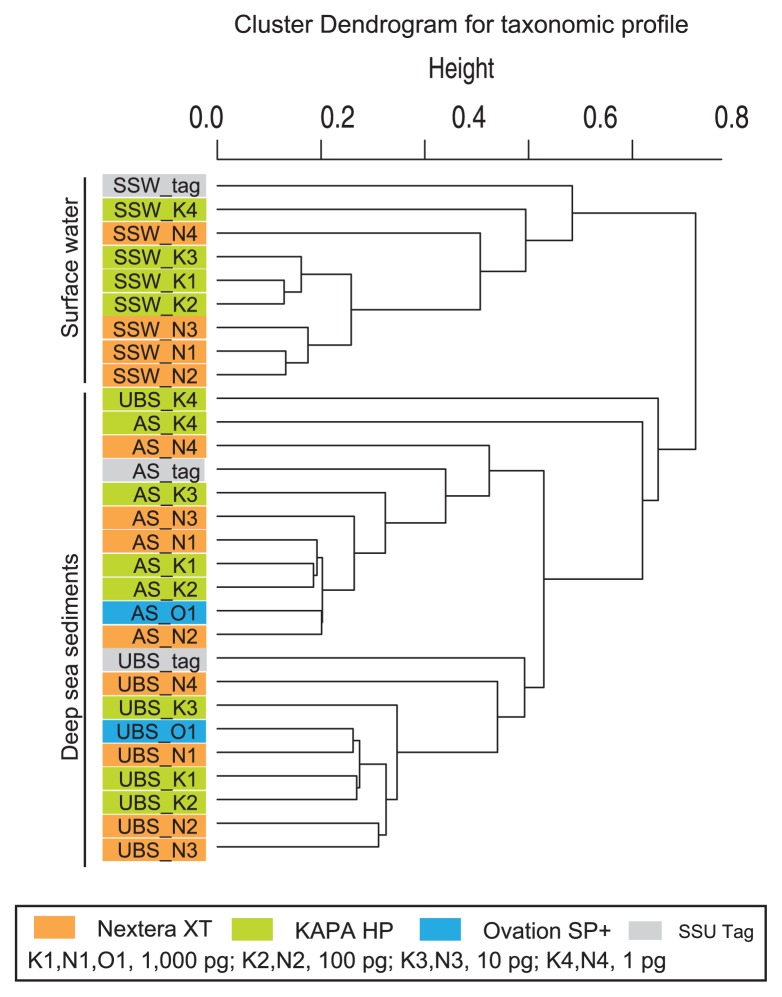
Clustering analysis of SSU rRNA gene tag communities and SSU rRNA gene sequence communities, identified from metagenomic libraries obtained from environmental DNA assemblages extracted from upper bathyal sediment (UBS: MR12E02_M840_0cm), abyssal sediment (AS: KR1401_A2_0cm), and subarctic surface water (SSW: MR1404_22_0m).

Reads encoding part of an SSU rRNA gene were identified and characterized to assess the microbial diversity in each shotgun metagenomic library (Fig. 3 and Table S1); a clustering analysis for SSU rRNA gene communities was also performed (Fig. 4). In addition, SSU rRNA gene tag sequencing was conducted as a reference for comparisons to SSU rRNA gene communities and the identification of potential contaminants during library construction (Figs. 3 and 4 and Table S3). The analyses presented three DNA assemblage-harbored genomes from three domains of life. The impacts of the potential contaminants on SSU rRNA gene compositions were limited in both metagenomic libraries and tag sequencing (Fig. 3, Tables S1 and S3), whereas it was apparent that the population of potential contaminants increased in the Nextera XP libraries constructed from 1 pg DNA. The SSU rRNA gene communities of the upper bathyal sediment (UBS: MR12E02_M840_0cm) and subarctic surface water (SSW: MR1404_22_0m) were dominated by *Bacteria* and *Eukarya*, whereas those in the abyssal sediment (AS: KR1401_A2_0cm) were dominated by *Archaea* and *Bacteria*. SSU rRNA gene community compositions were generally similar to each other in each DNA assemblage. However, the SSU rRNA gene community composition of metagenomic libraries constructed from 1 pg DNA were biased from those constructed from 10 to 1,000 pg DNA (Figs. 3 and 4). In the cluster analysis for SSU rRNA gene communities, the branch lengths between the metagenomic libraries constructed from 1 pg DNA and the libraries constructed from 10 to 1,000 pg DNA were similar to those between the SSU rRNA gene tags and the libraries constructed from 10 to 1,000 pg DNA (Fig. 4). This was also confirmed in the NMDS analysis (Fig. S5).

## Discussion

The present results show the inhibition of chemical contaminants associated with environmental DNA assemblages in enzymatic reactions in the KAPA HP kit and tagmentation in the Nextera XT kit, and a magnet bead purification system effectively resolved this issue. The effects of chemical contaminants, possibly humic substances, on PCR amplification have long been discussed for sediment samples (29, 30), and the successive application of a magnet bead purification system after conventional DNA purification was also reported (19). In the case of the subarctic surface water microbial community, we did not conduct size fractionation between prokaryotic and eukaryotic cells. In some of the previous metagenomic studies on planktonic prokaryotes including Rinke *et al.* (23) who examined the lower limit of input DNA for the Nextera XP metagenomic library construction, pre-filtration was applied to remove eukaryotic cells. They did not report the effects of chemical contaminants on library construction. In contrast, in the case of environmental studies on eukaryotic phytoplankton communities, the inhibition of chemical contaminants, possibly polysaccharides, on PCR has long been discussed (6, 32). The SSU rRNA gene community compositions of subarctic surface water showed a relatively high abundance of eukaryotic cells (Fig. 3). Accordingly, these results suggest that a magnet bead purification system needs to be applied for the metagenomic library construction of diverse environmental DNA obtained from a wide range of marine water and sediment samples.

Although Rinke *et al.* (23) suggested a lower limit of 1 pg using Nextera XT, the present results indicate that a threshold of low biased library construction is found between 1 and 10 pg starting DNA fo Nextera XT and KAPA HP. The larger bias in the libraries constructed from 1 pg starting DNA may be a result of more PCR cycles and/or inefficient linker ligation or tagmentation because of an insufficient substrate concentration. Nextera XT is known to have advantages in compositional similarity to the original starting mock microbial DNA over KAPA HP (11), as in the case of comparisons to other linker ligation-based library construction systems (14). However, as Jones *et al.* (11) noted, standardization of the library construction system is not feasible because technology continues to advance, the amounts and availability of microbiome samples are highly variable, and scientific budgets are constrained. This study using environmental DNA assemblages showed several advantages of KAPA HP over Nextera XT. The GC distribution of sequence reads revealed the lower coverage of AT-rich regions (>70%) in Nextera XT libraries than in KAPA HP libraries, as described above (Fig. 1). Less contaminated libraries were constructed using KAPA HP with a lower amount of DNA than Nextera XT (Fig. 3 and Table S1), and this may be due to the shorter procedure for KAPA HP; some of the classical library construction kits based on the physical fragmentation and linker ligation technique require longer working times. The present results suggest that 10 pg of input DNA is sufficient for constructing low biased shotgun metagenomic libraries using KAPA HP and Nextera XT with modified protocols, and will expand options for metagenomic library construction from small amounts of environmental DNA assemblages.

Moreover, read abundance, GC contents, and *k*-mer clustering analyses suggest that the GC contents and *k*-mer features of sequence libraries were primarily influenced by library construction methods (physical fragmentation and ligation vs. tagmentation), and the amount of starting DNA also affected these parameters (Figs. 1 and 2 and Table 1). The impact of library construction technologies on *k*-mers and the GC distribution of sequence reads was larger than that for the SSU rRNA gene sequence population. This result also suggests a risk of the over-interpretation of differences and similarities and the necessity for comparisons with multiple parameters among metagenomic libraries constructed using different technologies.

## Supplementary Material



## Figures and Tables

**Table 1:** Read quality and GC contents of shotgun metagenomic libraries.

Library ID	Input DNA (pg)	Library construction kit	PCR cycles	Library conc. (nM)	Sequenced reads	Low quality reads (%)	Low complexity reads (%)	Duplication reads (%)	Processed reads (%)	GC Read abundance*	

										Average GC%	Peak positions
**MR12E02_M840_0cm: Upper bathyal sediment (UBS) from off the Tohoku region (38.4980 N 142.3409 E, 845 m)**											
MR12E02_M840_0cm_K1	1,000	KAPA HP	14	64.03	2,799,226	11.26	0.01	0.02	88.74	53.2%	56.5%
MR12E02_M840_0cm_K2	100	KAPA HP	18	30.14	2,551,950	16.95	0.02	0.09	83.05	53.4%	56.5%
MR12E02_M840_0cm_K3	10	KAPA HP	21	11.25	2,513,767	15.98	0.02	1.5	84.02	53.3%	56.5%
MR12E02_M840_0cm_K4	1	KAPA HP	25	2.87	2,108,350	36.25	0.4	6.66	63.75	53.7%	57.5%
MR12E02_M840_0cm_O1	1,000	Ovation SP+	11	7.52	3,036,979	20.6	<0.01	0.21	79.4	52.3%	56.5%
MR12E02_M840_0cm_N1	1,000	Nextera XT	12	26.30	2,210,354	18.93	0.01	<0.01	81.07	54.0%	55.5%
MR12E02_M840_0cm_N2	100	Nextera XT	14	7.93	2,860,624	11.87	<0.01	<0.01	88.13	57.0%	59.5%
MR12E02_M840_0cm_N3	10	Nextera XT	17	7.41	3,174,413	8.33	<0.01	0.05	91.67	56.3%	58.5%
MR12E02_M840_0cm_N4	1	Nextera XT	20	3.68	3,902,705	10.75	<0.01	0.91	89.25	56.2%	58.5%
**KR1401_A2_0cm: Abyssal sediment (AS) from the Mariana Trench region (11.7463 N 142.1087 E, 5838 m)**											
KR1401_A2_0cm_K1	1,000	KAPA HP	14	20.36	3,330,923	8.91	0.12	0.27	91.09	57.0%	35.5%, 61.5%
KR1401_A2_0cm_K2	100	KAPA HP	18	41.59	3,601,533	11.57	0.05	0.27	88.43	56.5%	35.5%, 61.5%
KR1401_A2_0cm_K3	10	KAPA HP	21	20.91	2,405,665	15.3	0.16	1.8	84.7	56.4%	35.5%, 61.5%
KR1401_A2_0cm_K4	1	KAPA HP	25	3.54	2,822,057	34.39	0.28	12.39	65.61	57.1%	36.5%, 60.5%
KR1401_A2_0cm_O1	1,000	Ovation SP+	11	5.9	3,429,835	22.72	0.01	0.28	77.28	55.2%	35.5%, 60.5%
KR1401_A2_0cm_N1	1,000	Nextera XT	12	18.36	2,950,863	10.65	0.01	0.01	89.35	53.7%	37.5%, 59.5%
KR1401_A2_0cm_N2	100	Nextera XT	14	9.65	3,072,133	10.55	<0.01	<0.01	89.45	58.8%	61.5%
KR1401_A2_0cm_N3	10	Nextera XT	17	4.80	3,451,066	8.6	<0.01	0.06	91.4	58.9%	61.5%
KR1401_A2_0cm_N4	1	Nextera XT	20	3.97	4,123,537	11.19	<0.01	0.85	88.81	58.0%	61.5%
**MR1404_22_0m: Surface water (SSW) from the subarctic North Pacific (44.0859 N 155.0153 E, 0 m)**											
MR1404_22_0m_K1	1,000	KAPA HP	14	19.78	3,641,322	12.45	0.03	0.34	87.55	42.9%	34.5%, 48.5%
MR1404_22_0m_K2	100	KAPA HP	18	23.95	2,928,383	16.23	0.07	0.33	83.77	43.2%	34.5%, 49.5%
MR1404_22_0m_K3	10	KAPA HP	21	13.16	2,932,866	20.14	0.05	6.08	79.86	43.9%	34.5%, 49.5%
MR1404_22_0m_K4	1	KAPA HP	25	1.62	3,555,563	55.01	0.05	33.49	44.99	46.1%	35.5%, 49.5%
MR1404_22_0m_N1	1,000	Nextera XT	12	6.71	2,868,815	14.84	0.03	0.01	85.16	46.5%	38.5%, 49.5%
MR1404_22_0m_N2	100	Nextera XT	14	2.08	3,626,261	9.03	0.01	0.06	90.97	49.5%	40.5%, 49.5%
MR1404_22_0m_N3	10	Nextera XT	17	0.74	4,261,060	10.06	0.01	1.37	89.94	48.0%	38.5%, 48.5%
MR1404_22_0m_N4	1	Nextera XT	20	0.89	3,967,406	15.09	<0.01	5.14	84.91	49.5%	46.5%, 66.5%

Percentages of low quality, low complexity, and duplication reads were estimated from the number of reads detected by Trimmomatic, PRINSEQ, and Picard, respectively.

* GC distribution of sequence reads are shown in Fig. 1.
