# Supplementary material for: Library Construction from Subnanogram DNA for Pelagic Sea Water and Deep-Sea Sediments
Source: Microbes Environ. 2017 Nov 28;32(4):336–43. doi: 10.1264/jsme2.ME17132 (PMC5745018; doi:10.1264/jsme2.ME17132)
Supplement: Supplementary file 1 [file 32_336_s1.pdf]

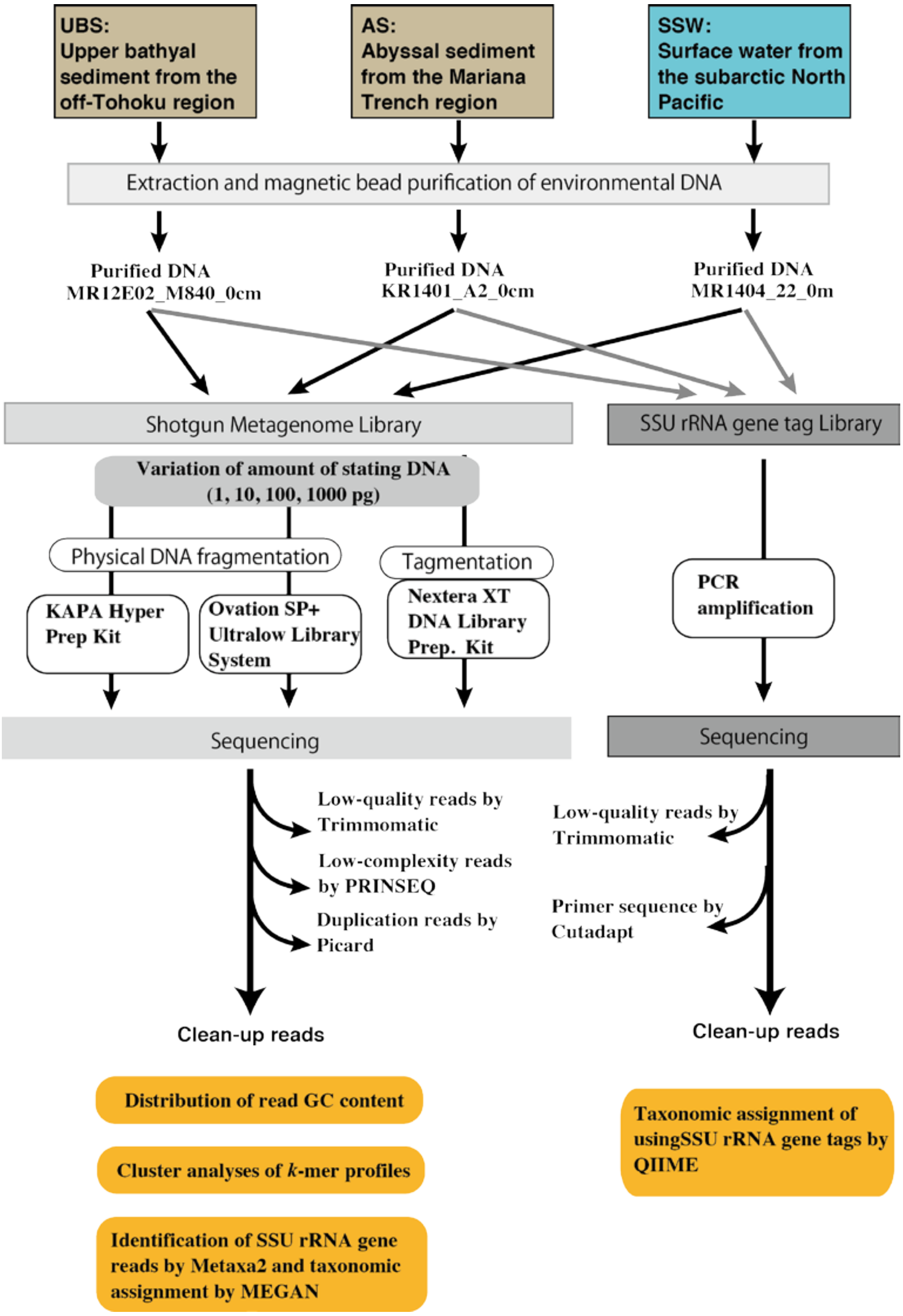

Fig. S1. Workflow of the DNA preparation, library construction and sequencing analyses.

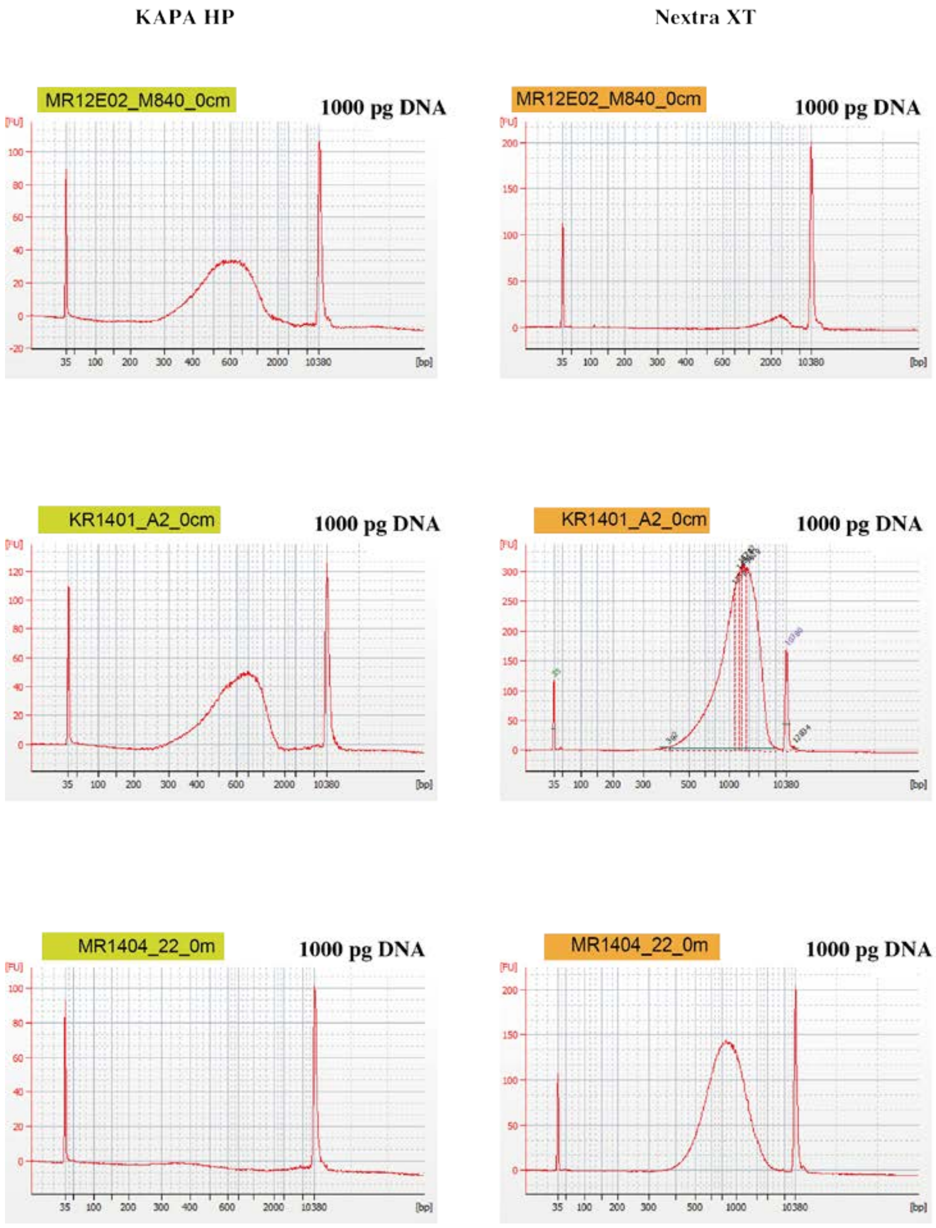

Fig. S2. Bioanalyzer electropherograms of the shotgun metagenomic libraries constructed from 1000 pg environmental DNA without magnetic bead purification using KAPA HP and Nextera XT kits.

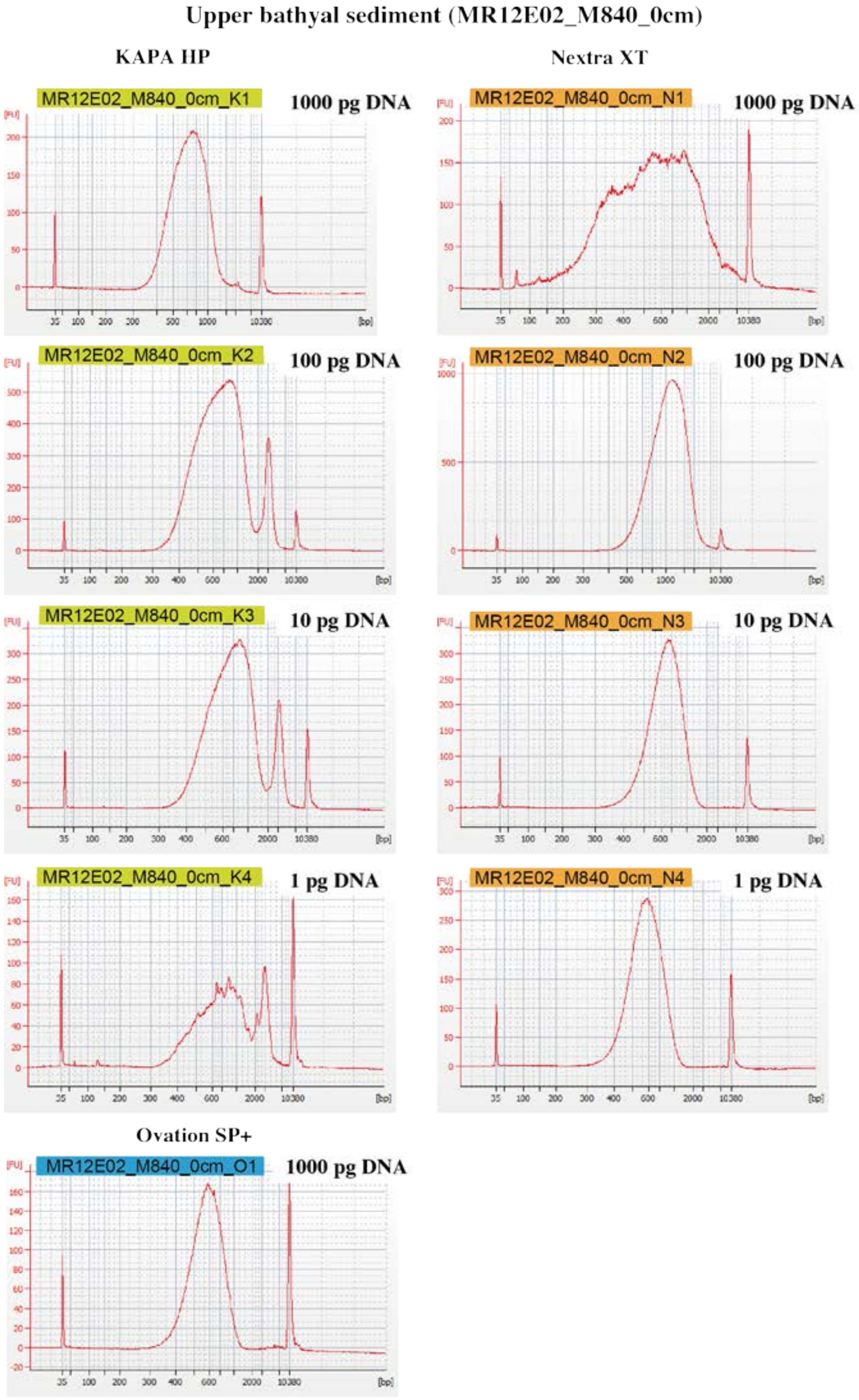

Fig. S3-1. Bioanalyzer electropherograms of the sequenced shotgun metagenomic libraries constructed from the environmental DNA using KAPA HP, Nextera XT and Ovation SP+ kits. The libraries constructed from 1000 pg DNA were constructed with the manufacturers' protocols, and those from 1 to 100 pg DNA were constructed with the modified protocols. The DNA was extracted from the upper bathyal sediment of the off Tohoku region.

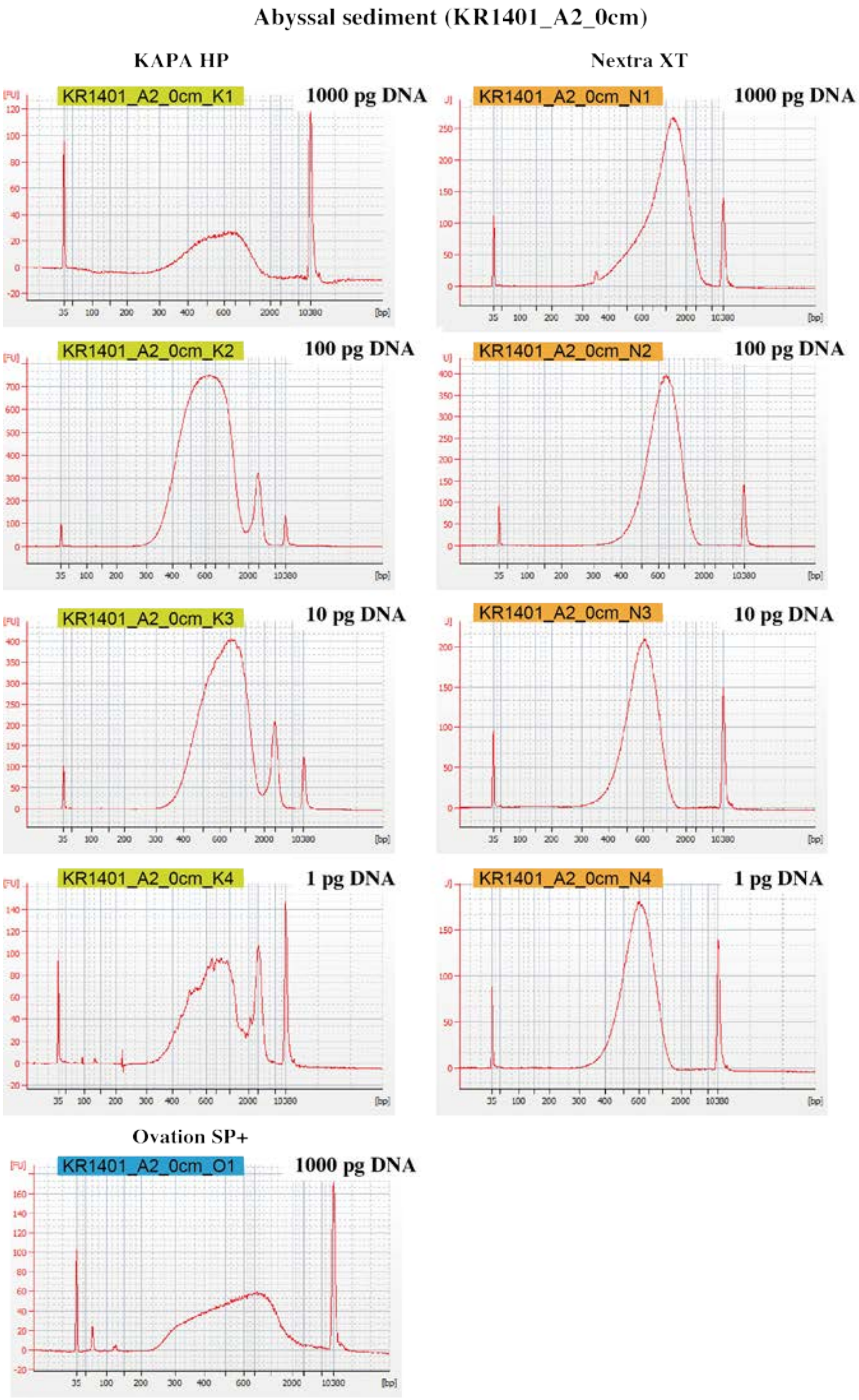

Fig. S3-2. Bioanalyzer electropherograms of the shotgun metagenomic libraries constructed from the environmental DNA using KAPA HP, Nextera XT and Ovation SP+ kits. The libraries constructed from 1000 pg DNA were constructed with the manufacturers' protocols, and those from 1 to 100 pg DNA were constructed with the modified protocols. The DNA was extracted from the abyssal sediment from the Mariana Trench region

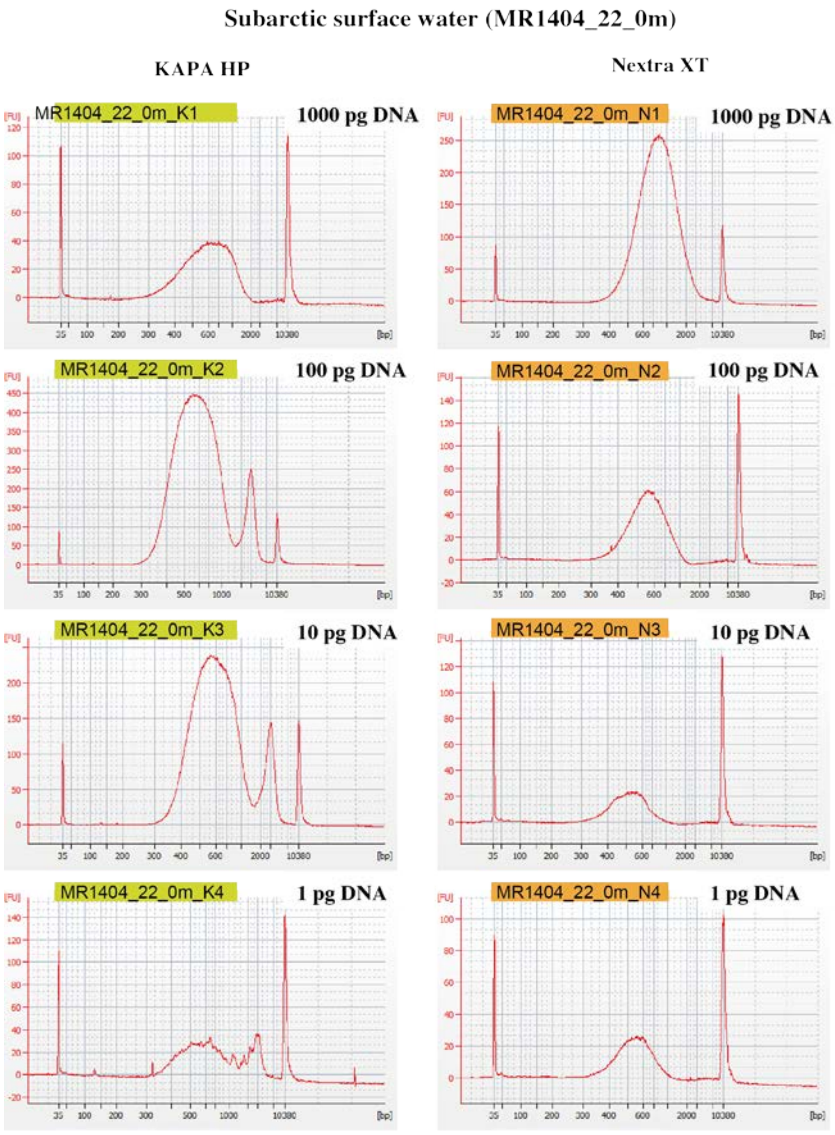

Fig. S3-3. Bioanalyzer electropherograms of the shotgun metagenomic libraries constructed from the environmental DNA using KAPA HP, Nextera XT and Ovation SP+ kits. The libraries constructed from 1000 pg DNA were constructed with the manufacturers' protocols, and those from 1 to 100 pg DNA were constructed with the modified protocols. The DNA was extracted from the subarctic North Pacific surface water

Upper bathyal sediment
(MR12E02_M840_0cm)

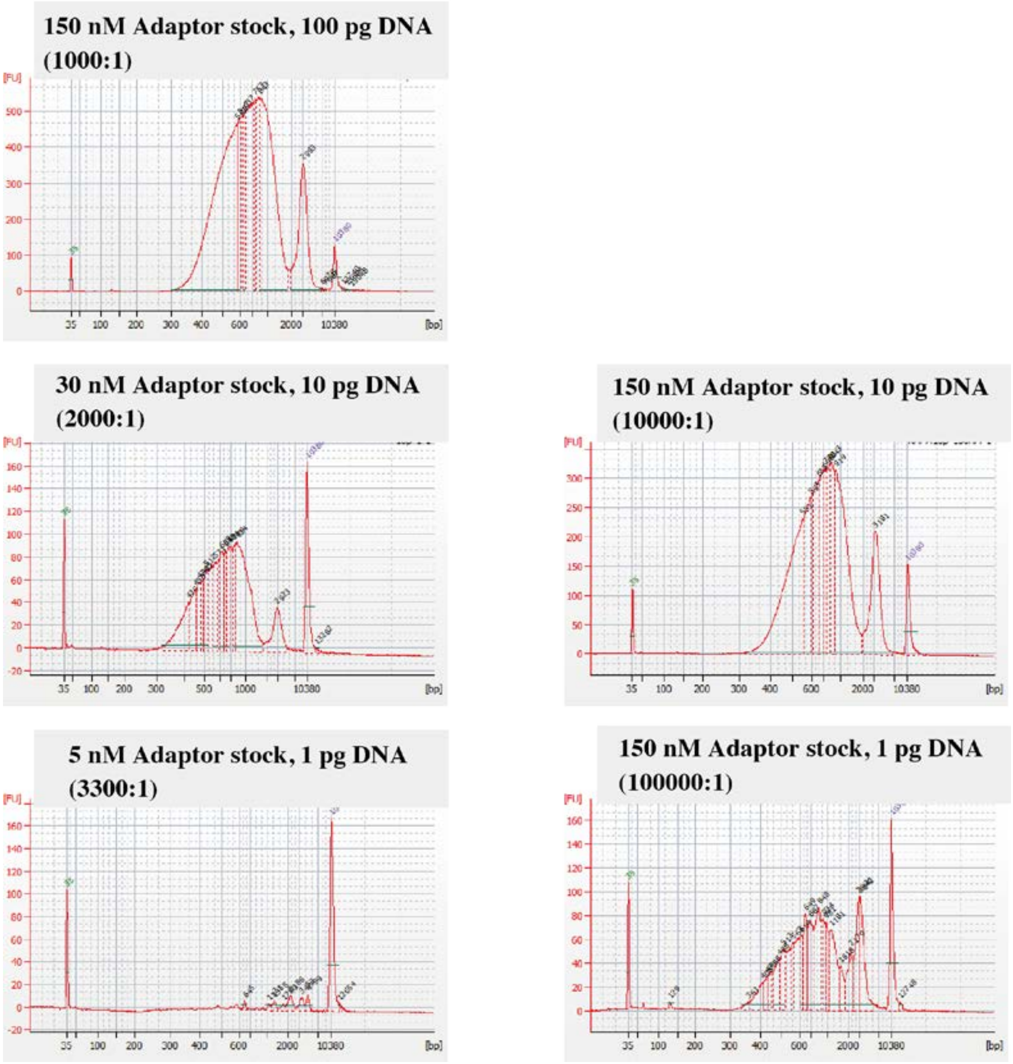

Fig. S4-1. Bioanalyzer electropherograms of the shotgun metagenomic libraries constructed from 1 to 100 pg DNA with adaptor stock concentrations of 5, 30 and 150 nM using KAPA HP kit. The DNA was extracted from the upper bathyal sediment.

Abyssal sediment
(KR1401_A2_0cm)

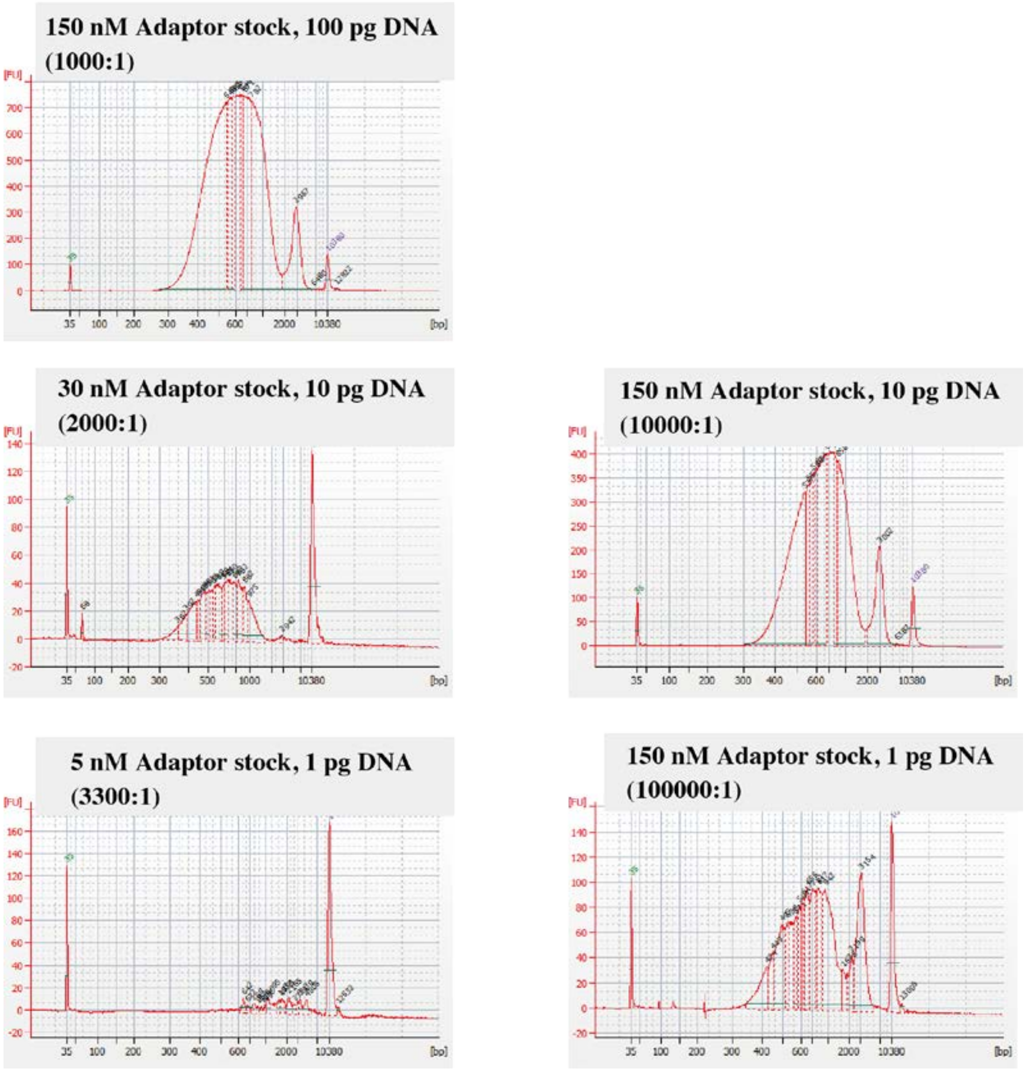

Fig. S4-2. Bioanalyzer electropherograms of the shotgun metagenomic libraries constructed from 1 to 100 pg DNA with adaptor stock concentrations of 5, 30 and 150 nM using KAPA HP kit. The DNA was extracted from the abyssal sediment.

North Pacific surface water
(MR1404_22_0m)

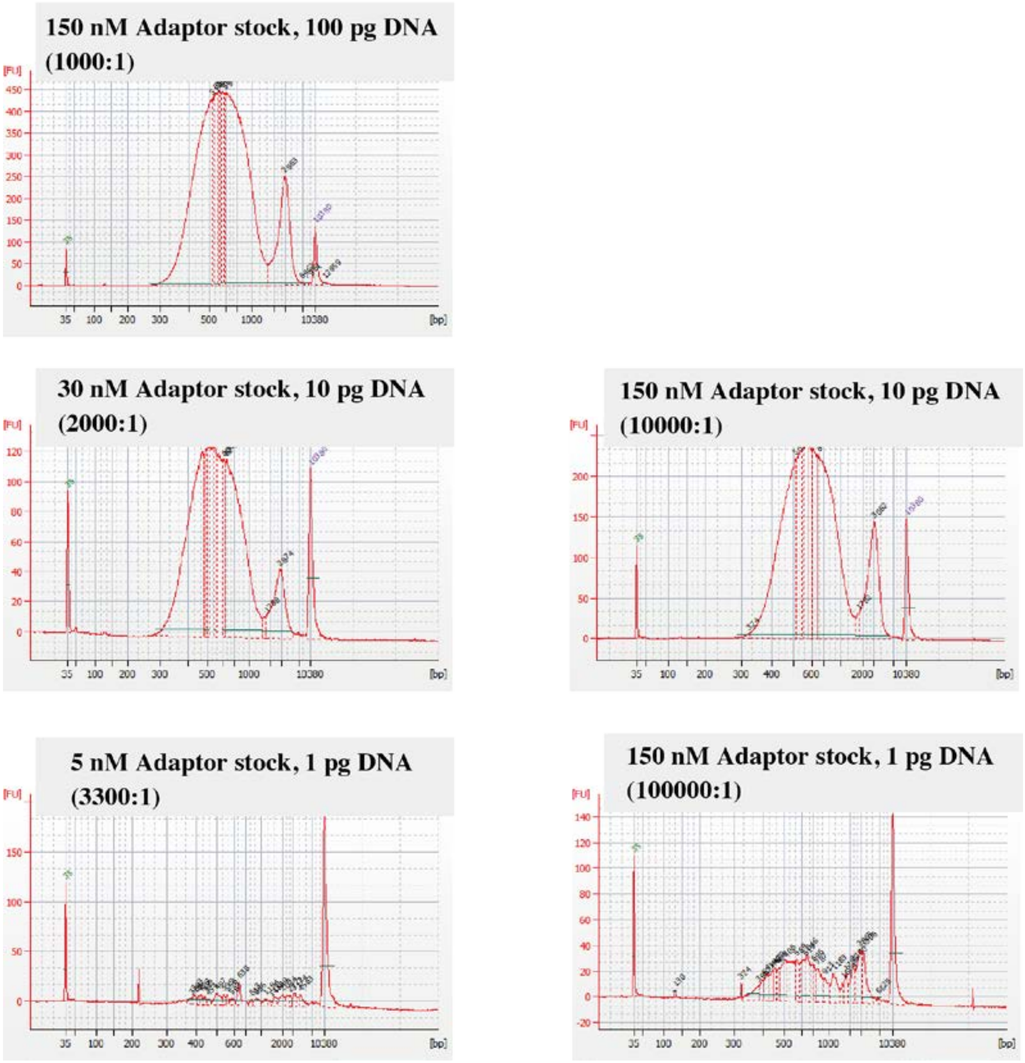

Fig. S4-3. Bioanalyzer electropherograms of the shotgun metagenomic libraries constructed from 1 to 100 pg DNA with adaptor stock concentrations of 5, 30 and 150 nM using KAPA HP kit. The DNA was extracted from the subarctic surface water

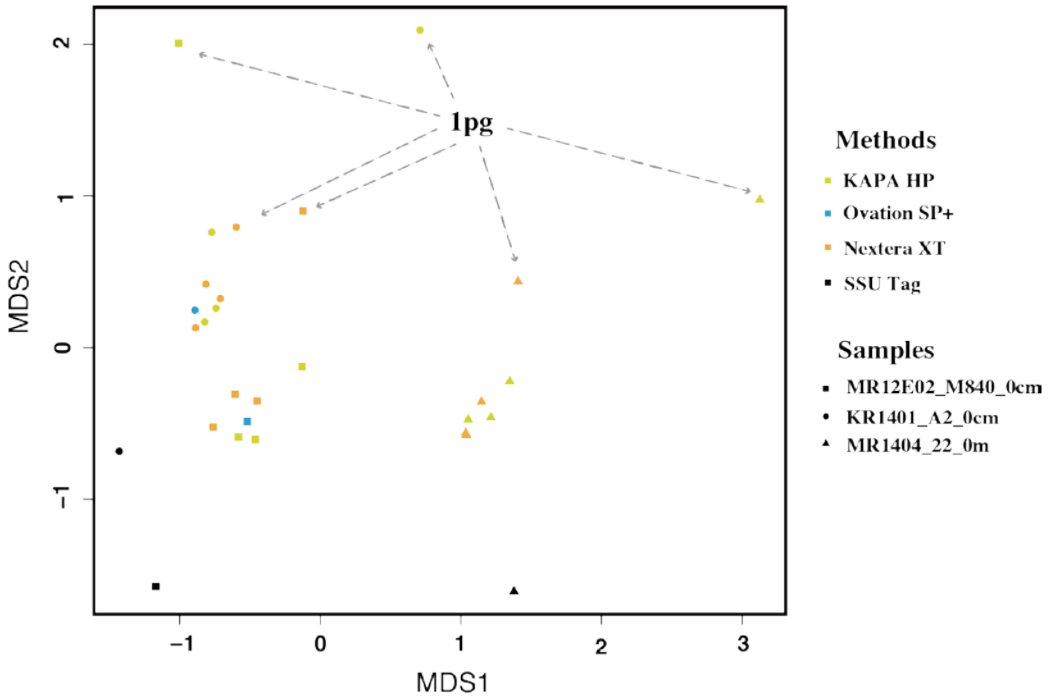

Fig. S5. Comparison of the SSU rRNA gene communities obtained from the shotgun metagenomic sequencing and the SSU rRNA gene tag sequencing by non-metric multidimensional scaling (NMDS) analysis.

Table S1. Composition of SSU rRNA gene sequences identified from the metagenomic libraries constructed from environmental DNA assemblages extracted from upper bathyal sediment, abyssal sediment and subarctic surface water.

			Upper bathyal sediment (MR12E02_M840_0cm)									Abyssal sediment (KR1401_A2_0cm)									Subarctic surface water (MR1404_22_0m)							
			KAPA HP				Ovation SP+	Nextera XT				KAPA HP				Ovation SP+	Nextera XT				KAPA HP				Nextera XT			
			MR12E02_M840_0cm K1	MR12E02_M840_0cm K2	MR12E02_M840_0cm K3	MR12E02_M840_0cm K4	MR12E02_M840_0cm O1	MR12E02_M840_0cm N1	MR12E02_M840_0cm N2	MR12E02_M840_0cm N3	MR12E02_M840_0cm N4	KR1401_A2_0cm_K1	KR1401_A2_0cm_K2	KR1401_A2_0cm_K3	KR1401_A2_0cm_K4	KR1401_A2_0cm_O1	KR1401_A2_0cm_N1	KR1401_A2_0cm_N2	KR1401_A2_0cm_N3	KR1401_A2_0cm_N4	MR1404_22_0m_K1	MR1404_22_0m_K2	MR1404_22_0m_K3	MR1404_22_0m_K4	MR1404_22_0m_N1	MR1404_22_0m_N2	MR1404_22_0m_N3	MR1404_22_0m_N4
Archaea	Euryarchaeota	Marine Group II	0	1	0	0	0	0	1	0	0	1	0	0	0	2	0	0	0	0	83	57	54	0	68	71	42	33
		Other Euryarchaeota	0	0	0	0	1	2	0	1	0	0	1	0	0	0	1	0	0	0	1	1	1	0	1	1	1	1
	Thaumarchaeota	Nitrosopumilus and relatives (MGI group a)	17	20	30	1	24	23	14	13	39	137	151	90	95	115	206	106	82	51	0	0	1	0	0	0	0	0
		Other Thaumarchaeota	0	0	2	0	0	0	0	1	1	72	73	51	1	73	145	83	75	129	0	0	0	0	0	0	0	0
	Woesearchaeota (DHVEG-6)		11	12	10	0	9	9	9	9	0	8	9	14	0	11	7	11	9	0	0	0	0	0	0	0	0	0
	Other Archaea		2	5	0	2	2	3	1	6	0	1	0	0	0	1	5	3	1	0	0	0	0	0	0	0	0	0
Bacteria	Acidobacteria	Holophagae	8	15	17	16	8	26	18	17	12	10	8	7	0	7	16	11	1	0	0	4	0	0	0	3	0	0
		Other Acidobacteria	21	17	9	0	23	21	32	41	39	61	58	59	42	66	63	57	70	139	0	0	0	0	0	0	0	0
	Actinobacteria	Acidimicrobiia	25	19	25	19	23	30	33	48	37	47	47	26	0	25	58	50	34	112	32	22	19	0	20	48	31	0
		other Actinobacteria *	2	3	0	0	3	3	5	1	46	1	1	1	0	3	3	1	5	51	0	1	0	0	3	4	8	128
		Armatimonadetes	0	0	0	0	0	0	0	4	64	2	2	0	0	0	1	0	5	43	0	0	0	0	0	0	7	152
	Bacteroidetes	BD2-2	3	0	0	35	3	0	0	1	9	0	0	0	0	0	0	0	0	0	0	0	0	0	0	0	0	0
		Cytophagia	15	14	19	30	15	14	14	9	21	27	24	7	0	23	20	18	7	6	3	5	0	0	7	7	2	0
		Flavobacteriia	32	25	50	3	31	24	20	14	9	4	8	4	0	1	7	4	2	0	163	168	148	85	367	379	473	264
		Sphingobacteriia*	8	8	10	0	6	4	3	5	26	2	2	0	0	3	3	0	1	0	3	3	0	0	7	9	1	0
		other Bacteroidetes	4	8	12	13	12	7	9	1	28	0	2	1	0	1	5	1	0	0	4	5	2	0	3	2	1	0
	OP3		11	3	8	0	8	3	6	4	2	12	11	6	0	4	12	11	15	23	0	0	0	0	0	0	0	0
	Chlamydiae		1	1	5	0	3	1	0	10	0	3	4	0	0	3	9	2	2	0	0	0	0	0	0	0	0	0
	Chlorobi		3	4	0	0	2	2	2	1	0	7	5	5	0	8	12	4	12	0	0	0	0	0	0	0	0	0
	Chloroflexi	SAR202	2	3	2	0	3	9	0	0	25	69	58	61	0	63	67	55	79	52	0	0	0	0	0	0	0	0
		Anaerolineae	9	5	12	0	10	8	10	3	39	8	9	10	0	10	15	12	24	0	0	0	0	0	0	0	0	0
		other Chloroflexi	12	8	10	1	8	8	12	12	19	38	36	37	5	25	40	54	60	97	0	0	0	0	0	0	10	31
	Cyanobacteria	chloroplast	9	8	7	0	5	6	1	4	0	0	0	0	0	0	0	0	0	0	270	181	241	5	226	305	261	213
		other Cyanobacteria	2	2	0	0	1	1	1	12	156	0	2	0	0	0	1	1	14	110	11	20	14	0	48	73	134	489
	Deferribacteres		4	0	1	0	0	2	0	6	0	8	4	9	84	14	2	3	2	23	0	0	0	0	0	0	0	0
	Marinimicrobia (SAR406)		4	4	2	0	1	1	1	3	0	5	6	3	0	2	2	2	1	0	15	12	7	0	12	19	16	1
	Elusimicrobia		4	2	0	0	0	0	3	5	0	1	1	0	43	1	5	4	6	0	0	0	0	0	0	0	0	0
	Fibrobacteres		6	4	3	0	2	2	1	6	0	1	0	0	0	1	1	0	0	0	0	0	0	0	0	0	0	0
	Firmicutes **		2	1	0	0	2	5	1	2	1	1	4	0	0	4	3	0	0	0	0	1	0	0	1	0	3	53
	Gemmatimonadetes		10	11	14	14	11	16	21	22	42	49	71	24	43	38	50	54	55	57	0	0	0	0	0	0	0	9
	Gracilibacteria		0	0	1	21	1	0	4	2	0	1	4	8	0	1	1	5	8	0	0	1	0	0	1	0	0	0
	Hydrogenedentes		4	3	5	0	5	7	3	1	0	3	9	2	40	6	4	7	9	2	0	0	0	0	0	0	0	0
	Latescibacteria		5	6	2	0	4	7	5	6	6	7	5	6	114	1	3	4	3	0	0	0	0	0	0	0	0	0
	Lentisphaerae		15	15	12	16	13	20	20	13	22	4	7	10	0	1	4	1	6	0	0	0	0	0	0	0	0	0
	Nitrospirae	Nitrospiraceae	8	6	7	0	7	5	4	14	0	8	14	1	0	10	12	9	14	0	0	0	0	0	0	0	0	0
		other Nitrospirae	0	0	0	0	0	0	0	0	0	2	3	3	5	3	0	0	3	0	0	0	0	0	0	0	0	0
	Parcubacteria		2	3	3	0	5	4	3	2	2	6	9	2	0	5	15	10	12	1	0	0	0	0	0	0	0	0
	Planctomycetes	Scalindua (Brocadiaceae)	8	10	9	0	12	13	10	20	6	0	0	0	0	0	0	0	0	0	0	0	0	0	0	0	0	0
		BD7-11	8	3	12	16	4	5	4	5	0	1	3	7	0	3	1	0	1	0	0	0	0	0	0	0	0	0
		OM190	15	16	7	0	12	13	13	9	0	14	13	20	0	6	14	6	2	42	0	0	0	0	0	0	12	0
		Phycisphaerae	17	11	8	16	17	21	20	17	0	70	84	28	103	63	76	69	61	94	0	0	0	0	0	0	0	0
		Planctomycetaceae	17	13	2	0	17	21	17	28	22	14	17	7	0	16	15	16	7	0	0	1	0	0	1	8	0	0
		other Planctomycetes	13	6	9	0	6	21	10	4	0	13	9	13	0	11	8	8	9	43	0	0	0	0	0	0	0	0
	Alphaproteobacteria	OCS116	0	1	0	0	0	0	1	5	0	1	0	0	0	0	0	0	0	0	16	9	0	0	7	23	11	11
		Rhizobiales**	7	2	8	0	5	1	2	22	0	4	3	1	0	6	8	11	6	21	7	5	0	0	13	15	21	0
		Rhodobacterales	8	11	7	0	4	8	12	4	11	13	18	5	2	12	21	10	14	0	199	176	192	77	368	494	499	558
		Rhodospirillales	15	10	21	0	10	13	11	10	15	64	71	56	0	63	71	73	77	55	137	88	99	0	104	139	57	15
		mitochondria (Rickettsiales)	3	5	6	1	0	0	0	0	0	0	1	0	0	0	0	0	0	0	34	30	26	0	22	41	36	26
		Rickettsiales	1	0	0	0	0	0	1	1	0	1	0	0	0	0	0	0	0	0	55	39	45	0	64	84	47	9
		S26-47	0	0	0	0	0	0	0	0	0	0	0	0	0	0	1	0	0	16	0	0	0	0	0	0	0	0
		SAR11	0	0	0	3	0	0	0	1	0	0	0	0	0	0	0	0	0	0	807	564	562	396	716	865	792	252
		Sphingomonadales*	0	0	0	0	0	0	0	1	2	0	0	0	0	0	0	0	0	0	0	1	0	0	1	5	0	0
		Caulobacterales*	0	0	0	0	0	0	0	0	0	0	0	0	0	0	0	0	0	0	1	0	0	0	0	2	7	0
		other Alphaproteobacteria	16	18	7	0	16	9	18	20	22	30	32	19	1	18	28	24	28	37	146	104	79	15	132	150	140	149
	Betaproteobacteria	Alcaligenaceae (Burkholderiales)	0	0	0	0	0	0	0	0	0	0	0	0	0	0	0	0	0	0	0	1	0	0	1	1	0	0
		other Burkholderiales*	0	0	3	14	0	0	0	0	0	0	0	7	0	0	0	0	0	0	1	1	3	0	2	2	1	0
		Methylophilaceae (Methylophilales)	0	0	0	0	0	0	0	0	0	0	0	0	0	0	0	0	0	0	15	14	17	0	11	12	9	0
		Neisseriaceae (Neisseriales)*	0	0	0	0	0	0	0	0	0	0	0	0	2	0	0	0	0	0	0	0	0	0	0	0	0	0
		Nitrosomonadaceae (Nitrosomonadales)	2	2	3	0	0	0	0	3	0	5	1	4	0	5	4	7	10	9	0	0	0	0	0	0	0	0
		other Betaproteobacteria**	3	3	1	0	2	0	3	0	1	1	0	1	0	1	2	3	2	0	20	7	8	0	12	14	6	0
	Nitrospinae	Nitrospinaceae	11	9	5	38	5	8	2	14	1	10	22	6	0	7	15	21	31	25	0	0	0	0	0	0	0	0
	Deltaproteobacteria	SAR324 (Marine group B)	3	3	3	9	6	6	4	4	0	14	5	11	10	8	12	10	6	4	0	0	0	0	0	0	0	0
		Bdellovibrionales	7	9	7	0	15	7	1	5	5	2	0	0	0	0	0	2	0	0	3	5	5	0	15	9	11	0
		Desulfobacterales	21	29	42	43	23	29	21	23	2	0	0	0	0	0	1	0	0	0	0	0	0	0	0	0	0	0
		Desulfuromonadales	4	3	6	22	5	2	1	1	0	2	1	0	0	1	0	1	0	25	0	0	0	0	2	5	1	0
		GR-WP33-30	0	0	0	0	2	2	2	0	0	7	11	4	0	9	13	12	5	0	0	0	0	0	0	0	0	0
		Myxococcales	41	23	19	0	26	46	36	53	12	6	3	1	0	3	4	3	7	2	0	0	0	0	1	0	0	0
		Oligoflexales	3	4	3	0	3	7	6	9	0	0	0	0	0	1	1	0	1	0	1	0	0	0	0	2	12	0
		Sh765B-TzT-29	65	49	51	4	40	54	74	85	34	48	49	29	0	49	64	62	97	100	0	0	0	0	0	0	0	0
		Sva0485	3	3	11	27	6	8	4	5	0	0	0	0	0	0	0	0	0	0	0	0	0	0	0	0	0	0
		DTB120	2	4	0	0	1	3	3	1	0	0	0	0	0	0	0	0	0	0	0	0	0	0	0	0	0	0
		Syntrophobacterales	5	5	0	0	2	12	11	7	0	0	0	0	0	0	0	0	0	0	0	0	0	0	0	0	0	0
		other Deltaproteobacteria	9	8	12	0	20	16	9	18	5	9	6	2	0	6	9	8	6	27	1	1	0	0	0	0	0	3
	Epsilonproteobacteria		1	1	0	0	2	0	0	0	0	2	0	2	0	0	0	0	0	0	1	0	0	0	0	1	0	0
	Gammaproteobacteria	Alteromonadales	6	8	1	0	5	6	9	7	0	4	1	1	0	1	0	1	0	0	4	3	2	0	8	16	2	0
		BD7-8 marine group	22	31	14	0	21	21	16	21	55	18	14	15	0	10	15	13	12	43	0	0	0	0	0	0	0	0
		Cellvibrionales	27	35	35	0	26	28	26	33	27	3	2	4	0	1	0	3	1	4	38	18	28	0	42	49	23	0
		Chromatiales	5	8	8	0	3	10	4	11	1	7	13	8	0	4	19	10	1	39	0	0	0	0	0	0	0	0
		Enterobacteriaceae (Enterobacteriales)*	1	0	3	2	0	0	1	0	0	0	1	7	1	0	1	0	0	0	3	2	7	0	2	1	0	0
		KI89A	0	5	0	0	4	3	3	0	2	6	16	4	0	4	6	5	6	0	5	8	4	0	10	10	4	30
		Legionellales**	1	0	0	0	1	3	0	2	0	1	0	1	0	0	2	0	0	0	0	0	0	0	2	0	0	0
		NKB5	0	0	0	0	2	0	0	4	0	1	0	0	0	0	1	0	0	0	0	0	0	0	1	1	13	0
		Oceanospirillales	12	12	35	31	9	9	16	15	33	5	8	0	0	3	3	3	7	0	581	378	362	71	401	445	333	80
		Pseudomonadales**	4	5	2	0	3	6	0	7	0	1	0	4	6	0	3	0	1	0	0	1	1	0	0	0	32	0
		Thiotrichales	1	3	0	0	0	2	1	0	0	0	0	0	0	0	0	0	0	0	19	11	6	0	16	12	2	0
		Xanthomonadales	54	48	57	33	58	52	64	62	103	86	106	63	21	74	102	84	78	44	1	0	3	0	2	0	1	0
		other Gammaproteobacteria	87	85	92	48	109	116	104	118	104	37	41	60	12	49	58	45	54	17	73	28	65	0	49	63	16	5
	JTB23 (Proteobacteria)		2	5	3	0	0	1	2	2	0	2	4	4	0	4	7	1	7	0	0	0	0	0	0	0	0	0
	other Proteobacteria		14	6	13	2	8	18	20	14	3	20	22	7	137	19	26	15	33	50	150	60	109	147	103	129	125	12
	TA06		1	1	3	0	0	0	1	5	3	6	6	0	0	4	7	1	2	0	0	0	0	0	0	0	0	0
	Verrucomicrobia		11	12	17	0	13	14	12	10	3	3	1	0	0	4	3	6	0	0	70	41	20	0	81	97	154	90
	other Bacteria		34	24	28	16	37	40	41	42	23	37	40	30	17	32	46	45	45	28	41	30	39	1	39	41	42	45
Eukarya	Archaeplastida		0	0	1	0	0	1	0	0	0	1	0	2	0	0	1	1	0	10	39	30	17	0	54	94	95	47
	Cryptophyceae		0	2	0	0	1	0	0	0	0	0	0	0	0	0	0	0	0	0	17	8	15	0	13	8	13	1
	Excavata		16	4	10	0	8	6	5	11	0	3	2	0	0	1	1	1	2	0	2	5	0	0	8	8	14	0
	Haptophyta		0	0	0	0	0	0	0	0	0	0	0	0	0	0	0	0	0	0	36	26	9	0	22	33	12	5
	Opisthokonta		226	178	196	40	200	173	94	130	184	2	3	4	0	6	5	2	5	5	600	470	433	589	956	1295	1172	839
	Alveolata		13	8	9	0	7	18	2	4	19	1	1	4	1	3	2	0	2	0	263	230	285	164	622	316	193	4
	Rhizaria		10	4	9	5	9	11	5	2	29	11	14	6	42	4	13	1	3	2	4	3	9	0	2	3	1	0
	Stramenopiles		11	3	11	0	8	9	5	5	0	0	0	0	0	0	1	0	0	0	99	56	58	0	88	111	122	24
	other Eukarya		8	7	5	0	7	12	4	7	2	8	4	0	0	10	10	6	2	7	25	24	12	0	50	28	40	5
	Total read count		1135	991	1092	541	1051	1157	988	1186	1374	1200	1296	894	827	1052	1498	1172	1248	1645	4099	2959	3007	1550	4807	5553	5061	3584
	Potential contaminant read count		28	22	27	16	22	22	15	40	76	11	11	22	9	17	25	15	15	72	35	22	19	0	43	52	79	181
	(% in total read count)		2.47	2.22	2.47	2.96	2.09	1.90	1.52	3.37	5.53	0.92	0.85	2.46	1.09	1.62	1.67	1.28	1.20	4.38	0.85	0.74	0.63	0.00	0.89	0.94	1.56	5.05

Number of reads encoding partial SSU rRNA gene sequences identified from the metagenomic libraries constructed from environmental DNA assemblages extracted from upper bathyal sediment, abyssal sediment and subarctic surface water. Numbers in parentheses indicate number of sequences identified in each library.

*, Taxonomic groups identified as potential laboratory contaminants.

**, Taxonomic groups that include sequences identified as potential laboratory contaminants.

Table S2 List of 7bp index in P5 and P7 primers.

P5	P7
GTGGTGG	TCCCTTG
CTCACAA	ACGAGAC
TAGTATG	GCTGTAC
ATGGCTG	ATCACCA
GTTCTCT	TGGTCAA
CGTAAGA	ATCGCAC
GCGTTCT	GTCGTGT
GTTGTTC	AGCGGAG
GGACTTC	ATCCTTT
TGCGCTG	TACAGCG
CTGCTAT	ACCGGTA
ATGTCAC	AATTGTG
TGTAACG	TGCATAC
AGCAGAA	AGTCGAA
TGGAGTA	GAATACC
GATCCCA	GTAGATC
TACCGCT	TAACGTG
TGTGCGA	CCAATAC
GATTATC	GATCTGC
GCCTAGC	CAGCTCA
GATGTAT	CAAACAA
ACTCCTT	GCGATAT
GTCACGG	GTATCTG
GCGAGCG	AGATTGA
ATCTACC	AGTTACG
ACTTGGT	TTGCGTT
TCTTGGA	TACGAGC
TCACCTC	GAGCCAT
GCACACC	AAGGCGC
GCGACAA	TGTGAAT
TCATGCT	CATTCGT
AGCTGTC	GGCCAGT
TACTCGG	ACTCACA
CGTGCTT	ATGATGA
CACTCAT	GTCGACA
TTGCCAA	AGGCTTA
GCAATTA	CTCACCT
CATACCG	CCACAGA
	CGTAATT
	AACTAGT
	AGCATGT
	GTACGAT

Table S3. Compositions of SSU rRNA gene tag communities in environmental DNA assemblages extracted from upper bathyal sediment, abyssal sediment and subarctic surface water.

			Upper bathyal sediment MR12E02_M840_0cm	Abyssal sediment KR1401_A2_0cm	Subarctic surface water MR1404_22_0m
Archaea	Euryarchaeota	Marine Group II	11	13	868
		Other Euryarchaeota	55	27	0
	Thaumarchaeota	Nitrosopumilus and relatives (MGI group a)	346	9180	5
		Other Thaumarchaeota	2389	13783	4
	Woesearchaeota (DHVEG-6)		1207	1054	0
	Other Archaea		192	29	0
Bacteria	Acidobacteria	Holophagae	965	454	0
		Other Acidobacteria	1387	2347	0
	Actinobacteria	Acidimicrobiia	852	1284	652
		other Actinobacteria	72	33	15
	Armatimonadetes	Armatimonadetes	12	39	0
	Bacteroidetes	BD2-2	373	7	0
		Cytophagia	1882	1308	209
		Flavobacteriia	3150	561	11740
		Sphingobacteriia	1163	361	317
		other Bacteroidetes	290	15	3
	OP3		446	762	0
	Chlamydiae		10	6	0
	Chlorobi		358	644	0
	Chloroflexi	SAR202	200	2747	1
		Anaerolineae	682	797	0
		other Chloroflexi	617	1371	0
	Cyanobacteria	chloroplast	478	8	8472
		other Cyanobacteria	185	58	935
	Deferribacteres		326	218	0
	Marinimicrobia (SAR406)		190	151	360
	Elusimicrobia		169	185	0
	Fibrobacteres		207	15	3
	Firmicutes **		189	73	2
	Gemmatimonadetes		819	2133	0
	Gracilibacteria		104	105	1
	Hydrogenedentes		304	302	0
	Latescibacteria		432	245	0
	Lentisphaerae		947	169	2
	Nitrospirae	Nitrospiraceae	342	319	0
		other Nitrospirae	7	77	0
	Parcubacteria		65	130	0
	Planctomycetes	Scalindua (Brocadiaceae)	150	0	0
		BD7-11	290	114	0
		OM190	921	805	4
		Phycisphaerae	1542	3263	3
		Planctomycetaceae	1793	1367	15
		other Planctomycetes	645	584	0
	Alphaproteobacteria	OCS116	117	13	598
		Rhizobiales**	309	243	175
		Rhodobacterales	505	521	7912
		Rhodospirillales	1322	4268	402
		mitochondria (Rickettsiales)	40	2	526
		Rickettsiales	176	72	2812
		S26-47	7	59	0
		SAR11	8	0	18041
		Sphingomonadales*	7	3	35
		Caulobacterales*	7	15	2
		other Alphaproteobacteria	348	100	159
	Betaproteobacteria	Alcaligenaceae (Burkholderiales)	1	0	14
		other Burkholderiales*	7	3	19
		Methylophilaceae (Methylophilales)	1	0	777
		Gallionellaceae (Nitrosomonadales)	97	0	0
		Nitrosomonadaceae (Nitrosomonadales)	5	558	0
		other Betaproteobacteria**	155	59	0
	Nitrospinae	Nitrospinaceae	759	1217	2
	Deltaproteobacteria	SAR324 (Marine group B)	509	505	5
		Bdellovibrionales	885	103	343
		Desulfobacterales	2357	17	0
		Desulfuromonadales	336	54	20
		GR-WP33-30	151	702	0
		Myxococcales	2486	364	6
		Oligoflexales	553	85	41
		Sh765B-TzT-29	2501	2639	0
		Sva0485	484	1	0
		DTB120	368	0	0
		Syntrophobacterales	757	4	0
		other Deltaproteobacteria	238	99	0
	Epsilonproteobacteria		30	0	0
	Gammaproteobacteria	Alteromonadales	262	48	153
		BD7-8 marine group	2509	1774	0
		Cellvibrionales	1913	125	964
		Chromatiales	1123	846	0
		Enterobacteriaceae (Enterobacteriales)*	2	1	1
		KI89A	185	231	212
		Legionellales**	150	148	9
		NKB5	191	62	12
		Oceanospirillales	882	207	7005
		Pseudomonadales**	42	1	30
		Thiotrichales	111	25	386
		Xanthomonadales	5190	5974	66
		other Gammaproteobacteria	4127	715	108
	JTB23 (Proteobacteria)		521	1148	0
	other Proteobacteria		390	237	115
	TA06		128	358	0
	Verrucomicrobia		378	159	1180
	other Bacteria		973	569	0
Eukarya	Archaeplastida		4	0	311
	Cryptophyceae		3	0	80
	Excavata		34	1	2
	Haptophyta		0	0	157
	Opisthokonta		2640	27	6114
	Alveolata		299	36	2102
	Rhizaria		234	31	21
	Stramenopiles		74	3	67
	other Eukarya		62	18	25
	Total read count		64717	71593	74620
	Potential contaminant read count		868	546	273
	(% in total read count)		1.34	0.76	0.37

The number in Table present read count corresponding the each taxon
*, Taxonomic groups identified as potential laboratory contaminants.
**, Taxonomic groups that include sequences identified as potential laboratory contaminants.
